# Tumour necrosis factor-α stimulates dehydroepiandrosterone metabolism in human fibroblast-like synoviocytes: a role for nuclear factor-κB and activator protein-1 in the regulation of expression of cytochrome p450 enzyme 7b

**DOI:** 10.1186/ar1819

**Published:** 2005-09-15

**Authors:** John Dulos, Allard Kaptein, Annemieke Kavelaars, Cobi Heijnen, Annemieke Boots

**Affiliations:** 1 Department of Pharmacology, Section Autoimmunity, N.V. Organon, Oss, The Netherlands; 2 Laboratory for Psychoneuroimmunology, University Medical Center Utrecht, Utrecht, The Netherlands

## Abstract

Glucocorticoids have successfully been used in the treatment of rheumatoid arthritis. Data suggest that 7α-hydroxy-dehydroepiandrosterone (7α-OH-DHEA), an immunostimulating metabolite of dehydroepiandrosterone, can block glucocorticoid-induced immune suppression. Formation of 7α-OH-DHEA is catalyzed by activity of cytochrome p450 enzyme 7b (Cyp7b). Recently, we reported that tumour necrosis factor (TNF)-α, IL-1α, IL-1β and IL-17 enhance Cyp7b mRNA expression and induce a concomitant increase in the formation of 7α-OH-DHEA by fibroblast-like synoviocytes (FLS) from rheumatoid arthritis patients. The aim of this study was to elucidate which signal transduction pathway is involved in the TNF-α-mediated induction of Cyp7b activity in FLS. We studied the effects of inhibitors of different signal transduction pathways on Cyp7b activity in FLS by measuring Cyp7b mRNA expression using reverse transcription PCR and by measuring the formation of 7α-OH-DHEA. We applied SN50, an inhibitor of nuclear translocation of transcription factors (i.e. activator protein-1 [AP-1] and nuclear factor-κB [NF-κB]); PSI, a proteasome inhibitor that prevents IκB degradation and thereby NF-κB release; SP600125, a c-Jun N-terminal kinase (JNK) inhibitor; and the mitogen-activated protein kinase inhibitors PD98059 (extracellular signal-regulated kinase) and SB203580 (p38). Cyp7b is constitutively expressed in RA FLS and can be activated in response to TNF-α. SN50 and PSI prevented the TNF-α-induced increase in Cyp7b activity, whereas the mitogen-activated protein kinase inhibitors PD98059 and SB203580 had no effect. In addition, inhibition of Cyp7b mRNA expression and activity was observed with SN50, PSI and SP600125, suggesting that NF-κB and AP-1 induce Cyp7b transcription. These findings suggest that NF-κB and AP-1 are involved in the TNF-α-enhanced formation of the dehydroepiandrosterone metabolite 7α-OH-DHEA. Our results are in accordance with presence of AP-1 and NF-κB binding sites in the Cyp7b promoter.

## Introduction

Rheumatoid arthritis (RA) is a chronic inflammatory disease characterized by hyperplasia of fibroblast-like synoviocytes (FLS), which is regarded to be important in cartilage and bone erosion [1]. Steroids such as dehydroepiandrosterone (DHEA), glucocorticoids, androgens and oestrogens have been shown to modulate the disease process in RA [2]. Several authors have suggested that the natural, abundantly present steroid DHEA may have immunostimulating effects [3,4]. Further data indicate that the 7α-hydroxy-dehydroepiandrosterone (7α-OH-DHEA) metabolite of DHEA, rather than DHEA itself, is responsible for these immunostimulating effects [5,6]. In several studies 7α-OH-DHEA was found to stimulate the immune system both *in vitro *and *in vivo*, and it has been suggested that 7α-OH-DHEA acts as an antiglucocorticoid [6,7].

The conversion of DHEA into 7α-OH-DHEA is catalyzed by cytochrome p450 enzyme 7b (Cyp7b) [8]. Because of the reported immunostimulating effects of 7α-OH-DHEA, we previously investigated the relation between Cyp7b activity and arthritis. We showed that the severity of murine collagen-induced arthritis was associated with an increase in Cyp7b activity and Cyp7b mRNA level in synovial biopsies [9].

Recently, we reported that Cyp7b mRNA expression and Cyp7b activity are present in FLS from patients with RA [10]. In addition, expression of Cyp7b in RA FLS was enhanced after *in vitro *treatment of these cells with tumour necrosis factor (TNF)-α, IL-1α, IL-1β and IL-17 [10]. TNF-α is abundantly produced in inflamed joints and is known to play a crucial role in the pathogenesis of RA [11]. Therefore, in the present study we used TNF-α to investigate which signal transduction pathway is involved in the TNF-α-mediated increase in Cyp7b activity in human FLS. Signaling pathways that mediate the effects of TNF-α include mitogen-activated protein kinases (MAPKs) and nuclear factor-κB (NF-κB) [12]. Three MAPK families have been implicated to play a role in RA, including extracellular signal (mitogenic)-regulated protein kinase (ERK)1/2; the stress-activated protein kinases, also called c-Jun NH_2_-terminal kinases (JNKs); and the p38 MAPKs [13]. The JNK pathway is of interest because of its capacity to phosphorylate the amino acids serine-63 and -73 on the c-Jun activation domain, which is a component of activator protein-1 (AP-1). AP-1 transcription factors consist of homodimers and heterodimers of the Jun and Fos family [14]. Apart from MAPKs, TNF-α activates nuclear translocation of NF-κB, which plays a central role in inflammatory diseases such as RA through induction of transcription of proinflammatory genes [15]. NF-κB is retained in the cytosol of nonstimulated cells by a noncovalent interaction with IκB. Upon stimulation by TNF-α, IκB is degraded and NF-κB is released and translocated to the nucleus inducing inflammatory gene expression [15].

Previous studies implicated a role for TNF receptor I in the regulation of Cyp7b activity [10], but these studies were inconclusive regarding the role played by TNF receptor II in regulation of Cyp7b activity. Thus, in order to study which signaling pathways are involved in TNF-α-induced Cyp7b activity, we used different inhibitors with relevance to TNF receptor signaling. SN50 was initially described as an inhibitor of nuclear translocation of NF-κB. However, in addition to its effect on NF-κB, SN50 blocks nuclear translocation of the AP-1 transcription factor [16,17]. For that purpose, the effect of SP600125 – a recently described inhibitor of JNK – on Cyp7b mRNA expression and activity was assessed [16]. The proteasome inhibitor PSI prevents degradation of IκB and thereby indirectly prevents NF-κB nuclear translocation [18]. To determine a possible role for MAPKs other than JNK in the TNF-α-induced Cyp7b activity, the ERK1/2 inhibitor PD98059 and the p38 inhibitor SB203580 were used.

In the present study we report that NF-κB and AP-1, but not ERK1/2 and p38, are probably involved in TNF-α-stimulated formation of 7α-OH-DHEA.

## Materials and methods

### Fibroblast-like synoviocytes

FLS cell lines were developed from synovial biopsies obtained from RA patients, after informed consent had been granted. All patients fulfilled the 1987 American College of Rheumatology criteria [19]. FLS were phenotyped as CD55^+ ^synovial fibroblasts, as described previously [20]. Briefly, the synovial tissue was minced and digested for 2 hours with 1 mg/ml collagenase A in Dulbecco's modified Eagle's medium (DMEM) at 37°C. The tissue homogenate was filtered through a fine sieve (200 μm), washed and cultured overnight in synoviocyte medium (Tebu-Bio, Heerhugowaard, The Netherlands) in 5% carbon dioxide and 37°C to allow separation of adherent cells from the nonadherent cell population. Nonadherent cells were separated and adherent cells were cultured further in synoviocyte medium. The cells morphologically presenting as FLS were used between passages 2 and 17 in the experiments.

### Antibodies and reagents

The anti-NF-κB-p65 was from Signal Transductions (Becton & Dickinson, Woerden, The Netherlands), and the biotinylated anti-mouse IgG antibody was from Brunschwig Chemie (Amsterdam, The Netherlands). TNF-α was bought from Peprotech (Tebu-Bio, Heerhugowaard, The Netherlands). The p38 MAPK inhibitor SB203580 and the ERK1/2-MAPK kinase (MEK)-1 inhibitor PD98059 were from Omnilabo (Breda, The Netherlands), dissolved in dimethylsulfoxide or methanol and used as controls. The proteasome inhibitor PSI and the JNK inhibitor SP600125 were purchased at Omnilabo (Breda, The Netherlands) and dissolved in dimethylsulfoxide. The SN50 peptide (Biomol, Plymouth, USA) was dissolved in DMEM/Ham's F-12 medium.

### Measurement of TNF-α-induced Cyp7b activity in fibroblast-like synoviocytes

In order to arrest cell growth, synoviocyte medium was replaced by DMEM/Ham's F-12 medium with 10% foetal calf serum (FCS) and the FLS were cultured for another 3 days in a 24-well plate (Greiner, Alphen a/d Rijn, The Netherlands). FLS were preincubated in the presence or absence of SN50 for 2 hours, or PSI, SP600125, SB203580, or PD98059 for 1 hour in 2% charcoal-treated (depleted from steroids) FCS. Charcoal-treated FCS were prepared by suspending charcoal (Norit A) in Tris buffer. The suspension was then centrifuged for 10 min at 8.000 N/kg, the supernatant was removed and FCS added to the residue. This suspension was stirred for 30 min at 45°C and the charcoal was removed by centrifugation for 10 min at 8.000 N/kg. The supernatant was sterilized by membrane filtration using filters of pore sizes 0.8 and 0.2 μm successively. Following heat inactivation, FCS was stored at -20°C until use.

FLS were incubated with or without TNF-α and 1,2,6,7-[^3^H]-DHEA (1.5 × 10E^-8 ^mol/l: NEN Life Science Products, Boston, MA, USA) for 24 hours. Steroid-containing medium (1 ml) was passed over a C18 Solid Phase Extraction cartridge (Sopachem, Wageningen, The Netherlands) to determine the conversion of 1,2,6,7-[^3^H]-DHEA into ^3^H-labelled 7α-OH-DHEA as a measure of Cyp7b activity. Steroids were eluted from the column with methanol. Next, ^3^H-labelled 7α-OH-DHEA and ^3^H-labelled DHEA were measured using high-performance liquid chromatography. The amount of 7α-OH-DHEA is expressed as the percentage of ^3^H-labelled 7α-OH-DHEA of the total amount of ^3^H-label measured. Recoveries after extraction were in the range 85–95%, and identification of 7α-OH-DHEA was confirmed by Gas Chromatography-Mass spectrometry GC-MS (data not shown).

### Detection of 7α-OH-DHEA levels by radioimmunoassay

To determine 7α-OH-DHEA levels in FLS, a radioimmunoassay was performed using antiserum against 7α-OH-DHEA. The 7α-OH-DHEA metabolite is formed by the activity of the enzyme Cyp7b. The radioimmunoassay was performed at the Institute of Endocrinology at Prague (Czech Republic) in cooperation with Dr R Hampl [21]. In brief, FLS were preincubated in the presence or absence of SN50 for 2 hours or PSI, SP600125, SB203580, or PD98059 for 1 hour in 2% charcoal-treated (depleted from steroids) FCS. Thereafter, FLS were incubated with or without TNF-α and 1.5 × 10E^-8 ^mol/l DHEA (Diosynth, Oss, The Netherlands) for 24 hours. Extraction was carried out using diethyl ether. Diethyl ether extracts containing 7α-OH-DHEA and 7β-OH-DHEA were evaporated under nitrogen, and the dry residue was dissolved in assay buffer and measured using radioimmunoassay as previously described [21].

### Immunohistochemistry of fibroblast-like synoviocytes

FLS were grown on chamber slides (Nalge Nunc International; Fisher Emergo, Landsmeer, The Netherlands) and preincubated for 2 hours in the presence or absence of SN50 (100 μg/ml or 200 μg/ml) and thereafter stimulated for 30 min with TNF-α (0.5 ng/ml). After washing with phosphate-buffered saline (PBS), cells were fixed in methanol for 10 min and dried. The samples were blocked with buffer containing 2% normal goat serum, 2% human serum, and 2% serum albumin in PBS/0.01% Triton X-100 (PBS/T) for 30 min. Cells were then incubated with anti-NF-κB p65 antibody in the same buffer for 1 hour at ambient temperature. After washing with PBS-T, the FLS were incubated for 45 min with biotinylated anti-mouse IgG. After washing, cells were incubated for 30 min with avidin-biotin-peroxidase (Brunschwig Chemie, Amsterdam, The Netherlands). Following washing, the substrate was incubated for 10 min with enhanced diaminobenzidine in stable peroxidase buffer (Pierce; Perbio Science, Etten-Leur, The Netherlands). Following extensive washing in milli-Q water and dehydration, coverslips were placed with Entellan (Merck, Amsterdam, The Netherlands) mounting medium. Slides were visually analyzed under a Nikon Alphaphot-2 microscope (Uvikon, Bunnik, The Netherlands).

### Cyp7b mRNA levels in fibroblast-like synoviocytes

FLS were preincubated with 200 μg/ml SN50 and then incubated in the presence or absence of TNF-α (0.5 ng/ml) for 6 hours. Cells were washed with PBS and total RNA was extracted with RNAzol (Campro, Veenendaal, The Netherlands). cDNA synthesis was done according to the manufacturer's protocol using random hexamer primers (Pharmacia, Woerden, The Netherlands) and reverse transcriptase (Pharmacia). For reverse transcription PCR, human Cyp7b sense (GTCCTGGAGAAATATTATGTGCAG) and antisense (CGCACACAGTAGTCCCCGG) primers were used. For GAPDH we used CCCTTCATTGACCTCAACTACATGG (sense) and GGTCCACCACCCTGTTGCTGTAGCC (antisense) as primers. Reverse transcription PCR was carried out using an Applied Biosystems (Nieuwerkerk a/d ijssel, The Netherlands) thermo cycler with an anneal temperature of 53°C.

### Computer analysis of the Cyp7b promoter region

The promoter sequence of the human Cyp7b gene was identified and exported from the Ensembl database (vs19.34b.2; 9 February 2004) using the MartView export function. As promoter region, -1,000 to +100 nucleotides were selected in relation to the transcription start site. Promoter analysis for transcription factor binding sites was performed using the GEMSLauncher version 3.6 from Genomatrix and MatInspector professional release 7 [22]. Core and matrix similarity settings were 0.75 and optimized -0.03, respectively. The transcription factor family matrices V$AP1F, V$NFAT, V$NFKB and V$STAT were used.

## Results

### SN50 inhibited TNF-α-stimulated Cyp7b expression and activity

**Figure 1:**
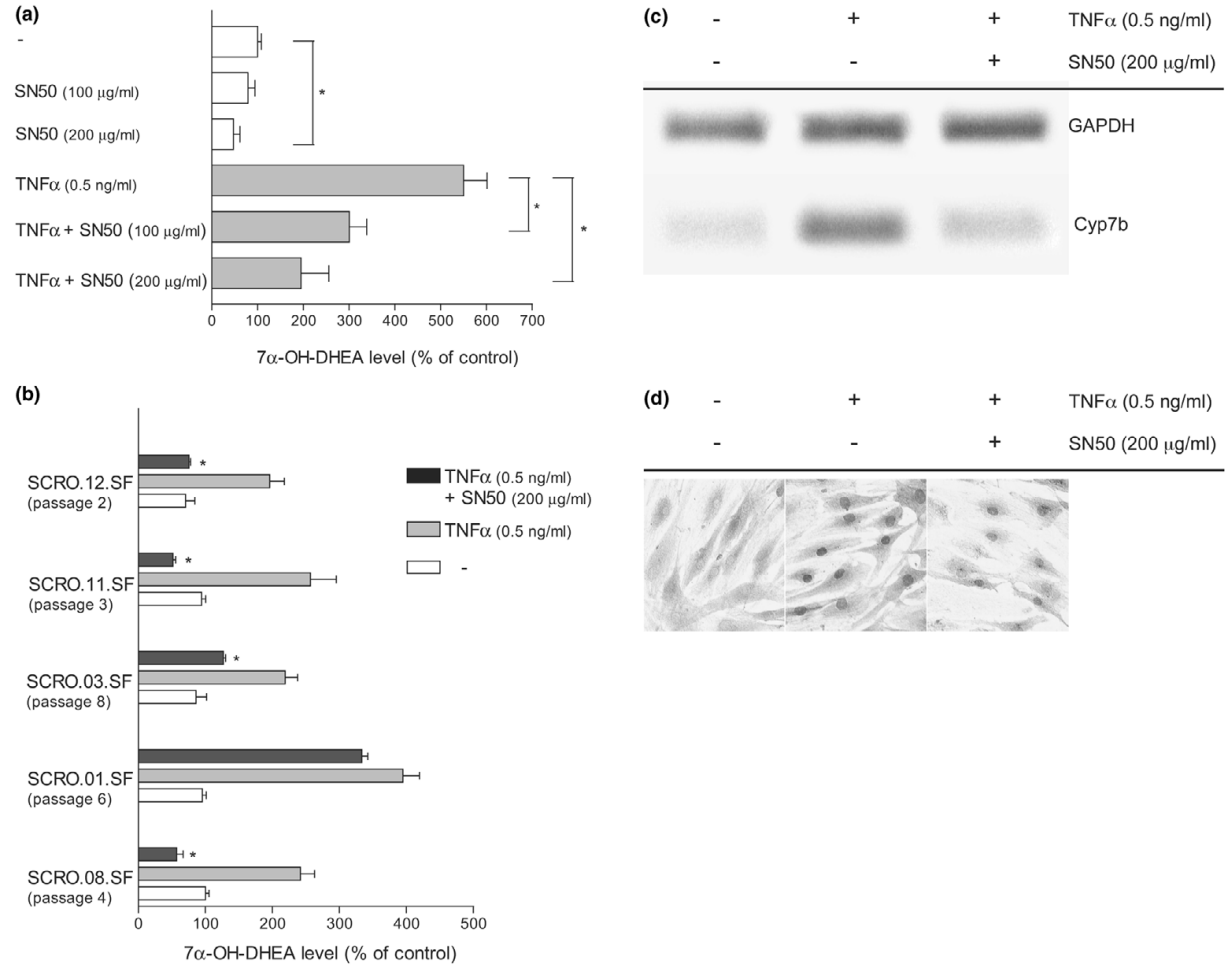
Cyp7b activity and mRNA expression is inhibited by SN50 in fibroblast-like synoviocytes. **(a) **Human fibroblast-like synoviocytes (FLS; SCRO.14.SF, passages 10–12) were plated at 1 × 10^5 ^cells/well in a 24-well plate and preincubated in the presence or absence (-) of the SN50 inhibitor for 2 hours. Thereafter, the cells were incubated with (solid bars) or without (open bars) tumour necrosis factor (TNF)-α for another 24 hours with 1.5 × 10^-8 ^mol/l ^3^H-dehydroepiandrosterone (DHEA). The formation of [^3^H]-7α-hydroxy-dehydroepiandrosterone (7α-OH-DHEA) from [^3^H]-DHEA, representing cytochrome p450 enzyme 7b (Cyp7b) activity, was determined by high-performance liquid chromatography. The amount of 7α-OH-DHEA is expressed as the percentage [^3^H]-7α-OH-DHEA of the total amount of [^3^H]-label measured. Results are expressed as the mean ± standard error of the mean of triplicate samples. The data are representative of two independent experiments. *P < 0.005 (Student's t-test). **(b) **Human FLS (1 × 10^5 ^cells/well) were isolated from five different rheumatoid arthritis patient biopsies. Cells (1 × 10^5^/well) were incubated in the presence and absence of TNF-α and in the presence of SN50 for 2 hours, as described in Materials and methods. Results are representative for one of the two independent experiments. SCRO.12.SF passage 2, SCRO.11.SF passage 3, SCRO.03.SF passage 8, SCRO.01.SF passage 6 and SCRO.08.SF passage 4 were used. *P < 0.005 versus TNF-α (Student's t-test). **(c) **FLS (SCRO.14.SF; passages 10–12) were incubated for 6 hours with 0.5 ng/ml TNF-α, SN50 200 μg/ml plus 0.5 ng/ml TNF-α, or incubated with medium control (-). Reverse transcription PCR was done using GAPDH and Cyp7b specific primers (35 cycles). The data are representative of two independent experiments. **(d) **FLS fibroblasts (SCRO.14.SF; passages 10–12) were grown on chamber slides. Cells were incubated for 2 hours in the presence or absence of 200 μg/ml SN50 before incubation for 30 min in the presence or absence of TNF-α (0.5 ng/ml). Immunoperoxidase staining was carried out with an antibody against nuclear factor-κB (NF-κB)p65 conjugated to peroxidase. Data are representative for three independent experiments.

An FLS cell-line (SCRO.14.SF), obtained from a synovial biopsy from an RA patient, was used to study the effect of SN50 on the TNF-α-induced Cyp7b activity. SN50 (200 μg/ml) significantly reduced basal Cyp7b activity (Fig. 1a). Importantly, the increase in Cyp7b activity following stimulation of the cells with TNF-α was dose-dependently inhibited by SN50 (Fig. 1a).

To further substantiate this finding, five other FLS cell lines generated from RA synovial biopsies obtained from different RA patients were stimulated with TNF-α with or without the dose of 200 μg/ml SN50. DHEA was metabolized into 7α-OH-DHEA in all five untreated FLS cell lines used (Fig. 1b). TNF-α induced a significant increase in Cyp7b activity in all FLS used. When SN50 was applied in combination with TNF-α, conversion of DHEA into 7α-OH-DHEA was significantly inhibited in four out of five FLS cell lines.

To investigate whether the effect of SN50 interfered at the level of Cyp7b activity or expression, we also analyzed the influence of SN50 on the TNF-α-induced increase in Cyp7b mRNA expression in the SCRO.14.SF cell line. A weak signal for Cyp7b mRNA was observed in untreated FLS (Fig. 1c). When stimulated with TNF-α, a marked increase in Cyp7b mRNA level was observed. Incubation of FLS with SN50 almost completely prevented the TNF-α-induced increase in Cyp7b mRNA expression (Fig. 1c).

Studies were performed to investigate whether SN50 indeed inhibits transport of NF-κB to the nucleus. In untreated FLS, NF-κB is localized in the cytoplasm (Fig. 1d). Incubation of FLS with TNF-α strongly increased the presence of NF-κB in the nucleus. This nuclear translocation of NF-κB was inhibited by SN50 (Fig. 1d).

### PSI inhibited the TNF-α-induced increase in Cyp7b activity

**Figure 2:**
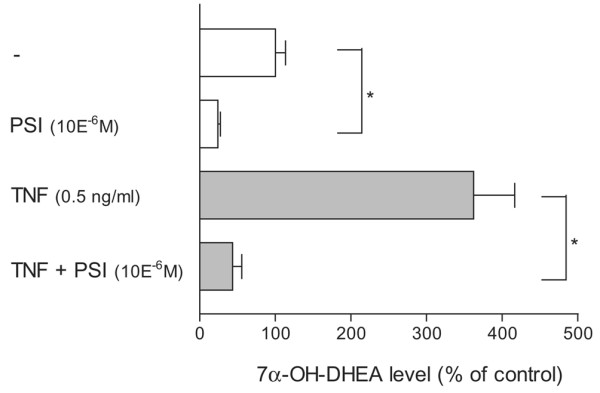
PSI inhibits the TNF-α-induced increase in 7α-OH-DHEA. Human rheumatoid arthritis (RA) fibroblast-like synoviocytes (FLS; SCRO.14.SF, 1 × 10^5 ^cells/well; passages 10–12) were preincubated in the presence or absence (-) of the PSI inhibitor for 1 hour. Thereafter, the cells were incubated with (solid bars) or without (open bars) tumour necrosis factor (TNF)-α for another 24 hours with 1.5 × 10^-8 ^mol/l ^3^H-DHEA, as described in Materials and methods. Data are expressed as mean ± standard error of the mean and are representative of four independent experiments. *P < 0.0005. 7α-OH-DHEA = 7α-hydroxy-dehydroepiandrosterone.

In subsequent experiments we examined the effect of PSI, a proteasome inhibitor that is known to prevent IκB degradation and thereby activation of NF-κB, on TNF-α-induced Cyp7b activation in the FLS cell line. PSI (1 × 10E^-6 ^mol/l) significantly decreased Cyp7b activity in nonstimulated FLS. Moreover, PSI prevented the increase in Cyp7b activity following incubation with TNF-α (Fig. 2). The combined results with SN50 and PSI imply an involvement of NF-κB in TNF-α-induced Cyp7b activity.

### MAPK inhibition did not affect the TNF-α-induced increase in Cyp7b activity

We further investigated a putative role for MAPKs in the TNF-α-induced increase in Cyp7b activity by using the MEK1 inhibitor PD98059 and the p38 inhibitor SB203580.

**Figure 3:**
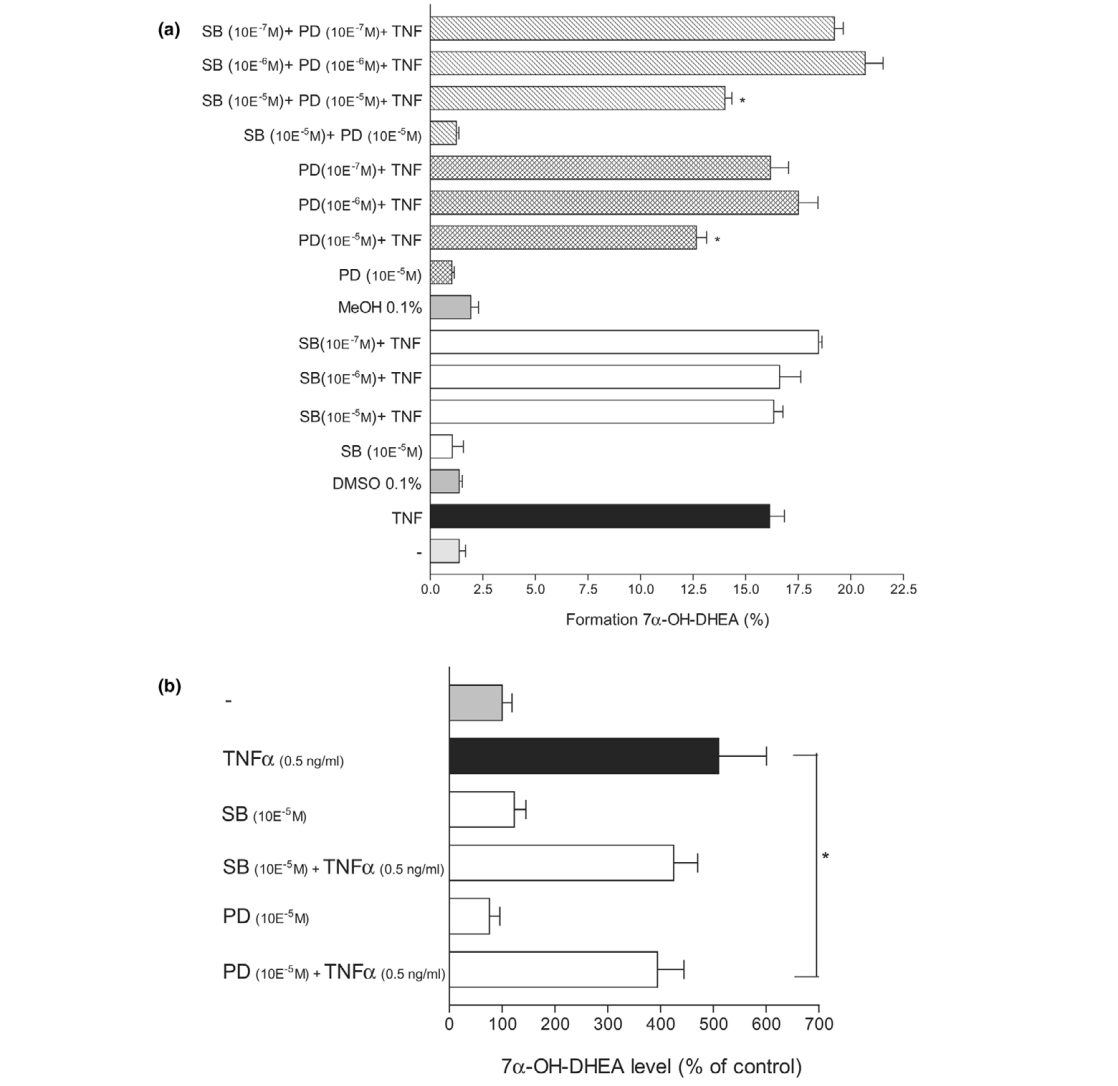
The effect of the MAPK inhibitors PD98059 or SB203580 on TNF-α-induced Cyp7b activity. **(a) **Fibroblast-like synoviocytes (FLS; SCRO.14.SF, passages 8–12) were incubated for 1 hour in the presence or absence (-) of the mitogen-activated protein kinase (MAPK) kinase (MEK)1 inhibitor PD98059 (PD) or the p38 inhibitor SB203580 (SB). Thereafter, cells were incubated in the presence or absence of 0.5 ng/ml tumour necrosis factor (TNF)-α plus 1.5 × 10^-8 ^mol/l [^3^H]-dehydroepiandrosterone (DHEA) for 24 hours and processed using high-performance liquid chromatography. The amount of 7α-hydroxy-dehydroepiandrosterone (7α-OH-DHEA) is expressed as the percentage [^3^H]-7α-OH-DHEA of the total amount of [^3^H]-label measured. Results are expressed as the mean ± standard error of the mean of triplicate sample. Data are representative of three independent experiments. *P < 0.05 versus TNF-α (Student's t-test). **(b) **The data from panel a (three independent experiments) are combined for the highest inhibitor concentrations. PD98059 and SB23580 were dissolved in methanol (MeOH) and dimethylsulfoxide (DMSO), respectively, and used as controls. *P < 0.05 (Student's t-test). Cyp7b = cytochrome p450 enzyme 7b.

The p38 inhibitor (SB203580) did not affect Cyp7b activity in nonstimulated cells (Fig. 3). Also, following TNF-α stimulation no effect of SB203580 on the increase in Cyp7b activity was observed. Similarly, incubation of nonstimulated FLS with the MEK1/ERK1/2 inhibitor (PD98059) did not affect Cyp7b activity. Only at a high concentration (1 × 10E^-5 ^mol/l) did application of PD98059 result in a small but statistically significant inhibition of TNF-α-induced increase in Cyp7b activity. The combination of SB203580 and PD98059 at high concentrations, similar to PD98059 alone, also exhibited a small but significant decrease in TNF-α-induced Cyp7b activity (Fig. 3). Similar findings were obtained using five additional RA FLS cell lines; a small inhibitory effect of the p38 inhibitor SB203580 at high concentration (1 × 10^-5 ^mol/l) was observed in one cell line out of five after stimulation with TNF-α. In none of the five cell lines did we observe any effect on the TNF-α-induced increase in Cyp7b activity using 1 × 10^-5 ^mol/l PD98059 (data not shown). From these results it is concluded that p38 and ERK1/2 do not appear to play a role in regulating Cyp7b activity.

### Regulation of Cyp7b mRNA expression and activity in fibroblast-like synoviocytes

**Figure 4:**
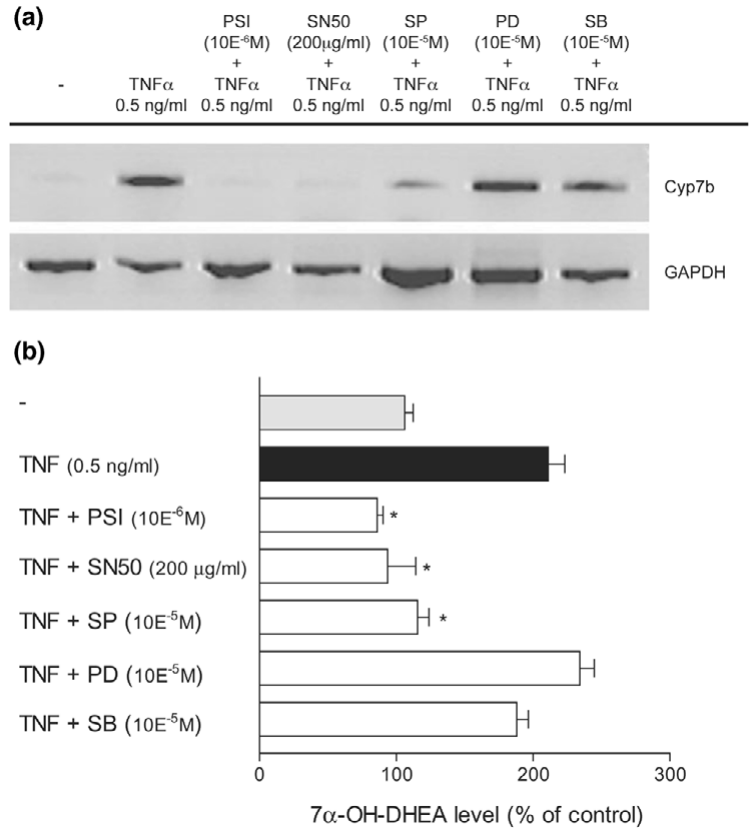
Regulation of Cyp7b mRNA expression and activity in FLS. **(a) **Human fibroblast-like synoviocytes (FLS; STSF.388, passages 8–10) were incubated for 6 hours with medium control (-), 0.5 ng/ml tumour necrosis factor (TNF)-α, TNF-α combined with the proteasome inhibitor (PSI) 1 × 10E^-6 ^mol/l, SN50 200 μg/ml, SP600125 (SP) 1 × 10E^-5 ^mol/l, PD98059 (PD) 1 × 10E^-5 ^mol/l or SB23580 (SB) 1 × 10E^-5 ^mol/l, as described in Materials and methods. RNA was isolated and cDNA was made and used for reverse transcription with GAPDH and cytochrome p450 enzyme 7b (Cyp7b) specific primers. The ratio of Cyp7b to GAPDH mRNA expression was 1.4 (TNF-α alone), 8 × 10^-6 ^(PSI + TNF-α), 1.3 × 10^-6 ^(SN50 + TNF-α), 0.5 × 10^-6 ^(SP + TNF-α), 0.3 (PD + TNF-α) and 0.2 (SB + TNF-α). Data are representative of two independent experiments. **(b) **FLS (STSF.388, passages 8–10) were preincubated in the presence or absence (medium control [-]) of SN50 for 2 hours or SP600125 (SP), PD98059 (PD) or SB23580 (SB) for 1 hour. Thereafter, FLS were incubated in the presence or absence of 0.5 ng/ml TNF-α plus 1.5 × 10^-8 ^mol/l dehydroepiandrosterone (DHEA) for 24 hours and processed for radioimmunoassay detection of 7α-hydroxy-dehydroepiandrosterone (7α-OH-DHEA). Results are expressed as the mean ± standard error of the mean of triplicate samples. Data are representative for two independent experiments. *P < 0.005 versus TNF-α (Student's t-test).

Previous studies implicated a role for TNF receptor I in regulating Cyp7b activity [10]. Because the TNF receptor I couples to AP-1 via the JNK pathway, we investigated the effect of the recently described JNK inhibitor SP600125 [17]. In addition, we analyzed the effect of NF-κB and MAPK inhibitors on TNF-α-induced Cyp7b mRNA expression. A weak Cyp7b mRNA signal was found in untreated FLS (Fig. 4a). Treatment of FLS with TNF-α resulted in an increase in Cyp7b mRNA expression. Moreover, SN50 prevented the increase in Cyp7b mRNA expression following incubation with TNF-α. Furthermore, the proteasome inhibitor PSI, which is known to prevent IκB degradation, blocked the TNF-α-induced Cyp7b mRNA expression. In addition, the JNK inhibitor SP600125 prevented the TNF-α-induced Cyp7b mRNA expression, which further substantiates a role for AP-1 in TNF-α-induced Cyp7b expression. Use of the MAPK inhibitors PD98059 and SB203580 did not result in convincing changes in TNF-α-induced Cyp7b mRNA expression.

We then determined Cyp7b enzymatic activity in FLS through the detection of 7α-OH-DHEA. Presence of TNF-α in the cultures resulted in increased Cyp7b activity compared with baseline (Fig. 4b). We subsequently analyzed the effect on TNF-α stimulation of the presence or absence of PSI, SN50, SP600125, PD98059 and or SB203580. TNF-α in combination with PSI, SN50, or SP600125 significantly decreased the Cyp7b activity to basal 7α-OH-DHEA levels (Fig. 4b). In contrast, addition of PD98059 or SB203580 did not significantly affect the TNF-α-induced increase in Cyp7b activity. The absence of an effect of the MAPK inhibitors PD98059 and SB203580 on TNF-α-induced Cyp7b activity is in accordance with our findings at the level of Cyp7b mRNA expression.

### Presence of NF-κB and AP-1 binding sites within the Cyp7b promoter

**Figure 5:**
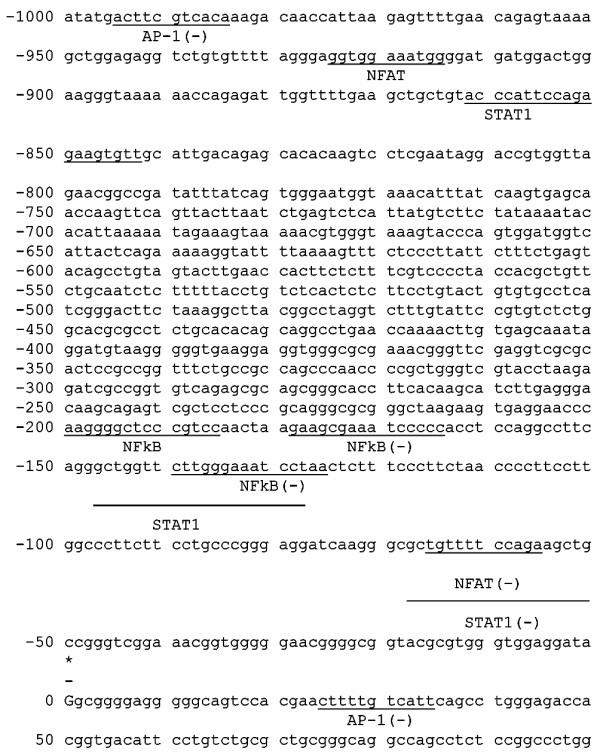
NF-κB and AP-1 binding sites within the Cyp7b promoter. Putative binding sites for selected transcription factor family matrices were identified using the MartView export function. Sequences for putative binding sites are underlined. *, transcription start side; -, presence of the transcription binding site on the minus DNA strand; AP-1, activator protein-1; Cyp7b, cytochrome p450 enzyme 7b; NFAT, nuclear factor of activated T cells; NF-κB, nuclear factor-κB; STAT, signal transducer and activator of transcription.

Analysis of the proximal region of the Cyp7b promoter revealed nucleotide sequences that correspond to putative binding sites for NF-κB, AP-1, nuclear factor of activated T cells (NFAT), and signal transducer and activator of transcription (STAT)1 (Fig. 5). The presence of putative bindings sites for NF-κB and AP-1 within the Cyp7b promoter are in accordance with the findings in this report that NF-κB and AP-1 are involved in the TNF-α-enhanced Cyp7b activity.

## Discussion

The findings of the present study suggest involvement of AP-1 and NF-κB, but not of p38 or ERK1/2, in the TNF-α-enhanced formation of the immunostimulating 7α-OH-DHEA.

We and others [23]. showed that, upon stimulation of cells with TNF-α, NF-κB translocates from the cytoplasm to the nucleus. As expected, translocation of NF-κB to the nucleus was inhibited by SN50. In addition, SN50 blocks the TNF-α-induced increases in Cyp7b activity and Cyp7b mRNA level, which suggests transcriptional involvement of NF-κB and/or other transcription factors such as AP-1 in TNF-α-induced Cyp7b activation. Initial reports suggested that SN50 is a specific inhibitor of NF-κB activation. However, Torgerson and coworkers [23] reported that SN50 blocks the nuclear translocation of the transcription factors AP-1, NFAT and STAT1 in Jurkat T cells stimulated with IFN-γ or phorbol myristate acetate (PMA) as well.

To determine whether STAT1 could be involved in TNF-α-induced Cyp7b activity, we analyzed the proximal region of the Cyp7b promoter for putative binding sites of STAT1, which revealed such sites in this region. It should be appreciated, however, that STAT1 is mainly activated by IFN-γ. Also, Cyp7b is not regulated by IFN-γ, as described previously [10]. Therefore, it is unlikely that STAT1 is involved in Cyp7b activity regulation.

There is evidence that the dose of SN50 determines the specificity of the inhibitor [16]. Therefore, it is likely that the doses of SN50 we used (100–200 μg/ml) can block both translocation of NF-κB and translocation of AP-1 to the nucleus [24]. Indeed, we observed inhibition of TNF-α-induced NF-κB nuclear translocation concomitantly with an inhibition of TNF-α-induced Cyp7b activity by SN50. In order to investigate a role for AP-1, we used the JNK inhibitor SP600125 [17]. The results demonstrate an involvement of the AP-1 complex in the TNF-α-induced Cyp7b expression and activity in FLS from RA patients. An involvement of NF-κB and AP-1 in the TNF-α-induced Cyp7b activity is in accordance with the presence of putative NF-κB and AP-1 binding sites within the Cyp7b promoter.

Our findings are consistent with data reported by Wu and coworkers [25] with respect to the presence of putative binding sites for NF-κB within the Cyp7b promoter. In contrast to our analysis, those authors [25] did not identify putative AP-1 binding sites, which could be due to the use of the default setting for the matrix score in MartView. However, other approaches are needed to substantiate further the role played by NF-κB and AP-1 in the TNF-α-induced increase in Cyp7B expression. This may be done by analysis of the Cyp7b promoter in a promoter reporter construct, with mutation of the putative NF-κB and AP-1 response elements. Moreover, the use of the siRNA technology could contribute to our understanding of the importance of NF-κB in the TNF-α-induced DHEA metabolism in human FLS.

Because the anti-glucocorticoid 7α-OH-DHEA, which is produced by the activity of the enzyme Cyp7b, might have stimulatory effects on the inflammatory process, studies with administration of 7α-OH-DHEA in animal models with susceptibility for arthritis are needed to elucidate the mechanism by which 7α-OH-DHEA influences the development of inflammatory processes. In this respect, it would be of interest to investigate whether inflammation is reduced in Cyp7b knockout mice, which do not express 7α-OH-DHEA. In addition, intra-articular delivery of 7α-OH-DHEA and/or Cyp7b expression systems should add to our understanding of the role played by Cyp7b in the arthritic process.

The inhibitory effect of PSI on the TNF-α-induced upregulation of Cyp7b activity is also in accordance with a role for NF-κB in regulating Cyp7b activity. Although it has not been described in the original studies of the action of PSI [18], we cannot exclude the possibility that inhibition of proteasome activity by PSI may interfere in other signal transduction pathways that are independent of NF-κB [26].

In this paper we show that inhibitors of the ERK1/2 and p38 signalling pathways did not convincingly affect Cyp7b mRNA expression and enzymatic activity in RA FLS following stimulation with TNF-α. Barchowsky and coworkers [27] also reported that there is no role for MAPKs after TNF-α stimulation of collagenase I expression in rabbit synovial fibroblasts. However, previous studies have reported activation of ERK1/2 and p38 in several cell lines, including synovial fibroblasts, after incubation with TNF-α [28]. We observed that, in contrast to TNF-α-induced Cyp7b activity, the MEK1/ERK1/2 inhibitor PD98059 and p38 inhibitor SB203580 reduced the TNF-α-induced IL-6 production in several RA FLS tested (data not shown). These results indicate that the inhibitors were active and can inhibit other effects of TNF-α, but they do not play a role in regulation of Cyp7b activity by TNF-α. Furthermore, it cannot be excluded that other MAPK isoforms such as ERK5, ERK7, p38γ and p38δ are regulated by TNF-α as well in the RA FLS used [29].

## Conclusion

**Figure 6:**
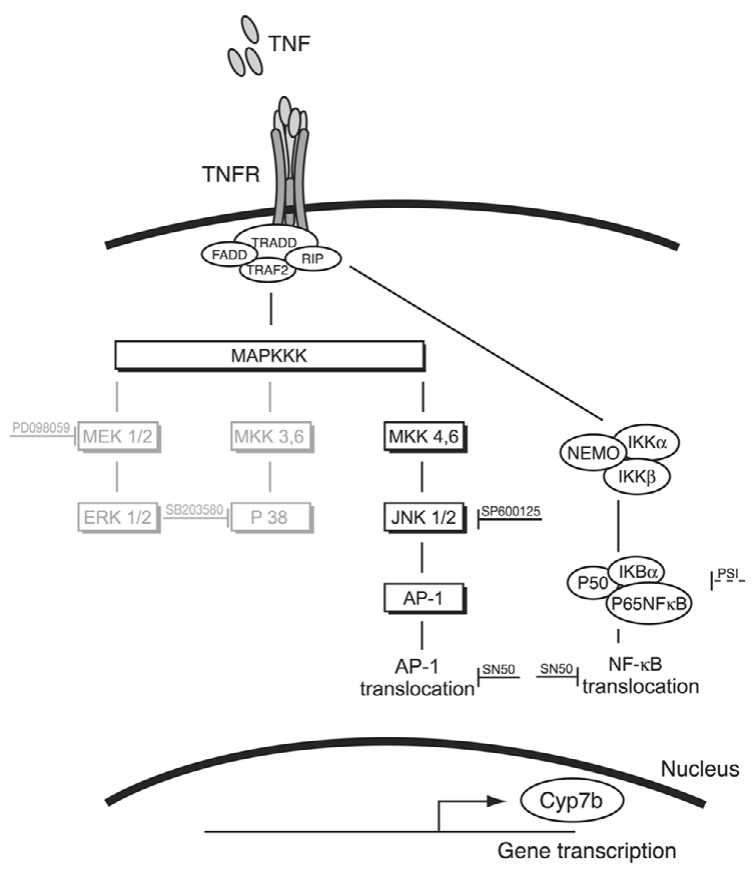
Simplified diagram of the proposed signalling events leading to Cyp7b gene transcription in synovial fibroblasts. Using inhibitors of the mitogen-activated protein kinase (MAPK) kinase (MEK)1/extracellular signal (mitogenic)-regulated kinase (ERK)1/2 pathway (i.e. PD98059), the p38 MAPK pathway (i.e. SB203580), the c-Jun-NH_2_-terminal kinase (JNK) pathway (i.e. SP600125), the IκB/nuclear factor-κB (NF-κB) pathway (i.e. PSI; dashed line) and the NF-κB/activator protein (AP)-1 pathway SN50, it was established that the NF-κB and AP-1 pathway is relevant to Cyp7b activity. All experiments were performed using synovial fibroblasts derived from patients with rheumatoid arthritis. Cyp7b, cytochrome p450 enzyme 7b; IκBα, inhibitor of NF-κB; IKK, IκB kinase complex (composed of three subunits – IKKα, IKKβ, and IKKγ [NEMO]; RelA (p65) and NF-κB1 [p50/p105] are subunits of NF-κB); RIP, receptor interacting protein; TNF, tumour necrosis factor; TNFR, TNF-α receptor; TRADD, TNF receptor associated death domain; TRAF, TNF receptor associated factor.

Our data suggest that there is a role for both NF-κB and AP-1 in regulating the expression and activity of Cyp7b (Fig. 6), which strengthens the rationale for specific inhibition of these pathways in arthritis.

## Abbreviations

AP-1 = activator protein-1; Cyp7b = cytochrome p450 enzyme 7b; DHEA = dehydroepiandrosterone; DMEM = Dulbecco's modified Eagle's medium; ERK = extracellular signal-regulated kinase; FCS = foetal calf serum; FLS = fibroblast-like synoviocytes; IFN = interferon; IL = interleukin; JNK = c-Jun N-terminal kinase; MAPK = mitogen-activated protein kinase; MEK = mitogen-activated protein kinase kinase; NFAT = nuclear factor of activated T cells ; NF-κB = nuclear factor-κB; 7α-OH-DHEA = 7α-hydroxy-dehydroepiandrosterone; PBS = phosphate-buffered saline; PCR = polymerase chain reaction; PMA = phorbol myristate acetate; RA = rheumatoid arthritis; STAT = signal transducer and activator of transcription; TNF = tumor necrosis factor.

## Competing interests

The author(s) declare that they have no competing interests.

## Authors' contributions

JD was principle investigator, and designed most of the studies, carried out most of the assays and wrote the manuscript. AK (Allard Kaptein) helped in conceiving the study and helped to draft the manuscript. AK (Annemieke Kavelaars) and CH were involved in drafting and revising the article. AB helped in conceiving the study, helped to draft the manuscript and was the senior scientist responsible for the work. All authors read and approved the final manuscript.
